# Income-related health inequalities across regions in Korea

**DOI:** 10.1186/1475-9276-10-41

**Published:** 2011-10-03

**Authors:** Eunju Hong, Byung Chul Ahn

**Affiliations:** 1 Department of Senior Industry Management, Hanyang Cyber University Haengdang 1 Dong, Seongdong Gu, Seoul 133-791, Korea; 2 School of Economics and Finance, Yeungnam University 280 Daehak-ro Kyungsan-si, Kyungbuk 712-749 Korea

**Keywords:** Bootstrapping, Gini Coefficient, Health Inequality, Convergence, Self-Rated Health, Concentration Index

## Abstract

**Introduction:**

In addition to economic inequalities, there has been growing concern over socioeconomic inequalities in health across income levels and/or regions. This study measures income-related health inequalities within and between regions and assesses the possibility of convergence of socioeconomic inequalities in health as regional incomes converge.

**Methods:**

We considered a total of 45,233 subjects (≥ 19 years) drawn from the four waves of the Korean National Health and Nutrition Examination Survey (KNHANES). We considered true health as a latent variable following a lognormal distribution. We obtained ill-health scores by matching self-rated health (SRH) to its distribution and used the Gini Coefficient (GC) and an income-related ill-health Concentration Index (CI) to examine inequalities in income and health, respectively.

**Results:**

The GC estimates were 0.3763 and 0.0657 for overall and spatial inequalities, respectively. The overall CI was -0.1309, and the spatial CI was -0.0473. The spatial GC and CI estimates were smaller than their counterparts, indicating substantial inequalities in income (from 0.3199 in Daejeon to 0.4233 Chungnam) and income-related health inequalities (from -0.1596 in Jeju and -0.0844 in Ulsan) within regions.

The results indicate a positive relationship between the GC and the average ill-health and a negative relationship between the CI and the average ill-health. Those regions with a low level of health tended to show an unequal distribution of income and health. In addition, there was a negative relationship between the GC and the CI, that is, the larger the income inequalities, the larger the health inequalities were. The GC was negatively related to the average regional income, indicating that an increase in a region's average income reduced income inequalities in the region. On the other hand, the CI showed a positive relationship, indicating that an increase in a region's average income reduced health inequalities in the region.

**Conclusion:**

The results suggest that reducing health inequalities across regions require a more equitable distribution of income and a higher level of average income and that the higher the region's average income, the smaller its health inequalities are.

## Introduction

Socioeconomic inequalities have represented one of the most controversial issues in Korea, mainly because Korea has achieved considerable economic growth much faster than most other countries, resulting in a substantially unequal distribution of income opportunities, education and health care resources, among others, within and between regions. For example, population density varies considerably across the country. The capital region (Seoul, Gyeonggi, and Incheon) accounts for only 11.8% of the land but accommodates approximately half of Korea's total population (48.2%; 22.8 million). In particular, Seoul (the capital) has a population of 9.8 million (20.8%), although it accounts for only 0.6% of the land. As of 2006, 51.8% of all manufacturers in Korea, which accounted for 48.5% of national employment, were based in the capital region [1]. Few large firms are headquartered outside the capital region, indicating a lack of attractive jobs available to local residents. Further, there exist substantial socioeconomic inequalities across regions outside the capital region, which have depended largely on differences in the level of industrialization across regions.

Such socioeconomic inequalities across regions have provoked a fierce policy debate searching for more equitability in various social dimensions, and thus, economic concentration, real estate bubbles, inequalities in education, and poor accessibility to medical resources/public health-care systems have been important buzz words in the media. The previous Rho administration (2003-2008) even initiated a policy initiative to relocate the capital and transfer some of the commercial resources and public enterprises from the capital region to underdeveloped regions to reduce the country's socioeconomic inequalities. This initiative was the subject of much political and legal debate. The competition among regions to secure limited resources and attract government corporations has been intensifying. Proponents argued that the policy would lessen regional conflicts and promote economic as well as social equality across regions. On the other hand, opponents brought this policy to the constitutional court, arguing that it was motivated more by political populism than by social justice and economic efficiency. After a long legal discussion, the court decided against the relocation of the capital but upheld the notion of spreading public organizations across the country.

In addition to the widespread economic inequalities that triggered the aforementioned political and legal debate, there has been growing concern over inequalities in public welfare, particularly those in inequalities in health across income levels and/or regions. Korea has witnessed substantial differences in health indicators across regions. For example, the mortality rate tends to be higher in low-income regions than in high-income ones [2]. Such differences, together with the aging population, have been a major source of concern over the public's health and access to adequate health care.

Health-care resources have been unevenly distributed across regions and highly concentrated in certain areas. As of 2009, Seoul accounted for 27.6% of medical specialists and 52.4% of physicians and dentists. Further, the capital region accounted for most of the high-tech medical equipment [3]. Consequently, medical expenditures are highly concentrated in the capital region. In 2008, Seoul accounted for 26.9% of all insured medical bills, and regions outside Seoul and the capital region accounted for 36.2% and 14.5%, respectively [4]. Between 2006 and 2008, half of the revenues generated by the top 20 general hospitals in Seoul were from patients residing outside Seoul [5]. This may be because hospitals in Seoul tend to be much better equipped than those in other regions and because Seoul has a more convenient and efficient transportation system.

This concentration can lead to the inefficient use of individual and national resources and exacerbate socioeconomic inequalities in health across regions. Previous studies have found income-related inequalities in health in various countries, including those in Europe [6,7], the U.K. [8], the U.S. [7,9], China [10-14], and Korea [15], among others. Thus, achieving income growth and reducing inequalities in health have become important national issues for many countries, including EU members [16]. Hence, various economic policies have been proposed to address these issues. In this regard, the present study examines whether an increase in regional income could reduce socioeconomic inequalities in health across regions.

Socioeconomic inequalities in health can be examined in many ways, including the concentration index (CI), which is an effective tool for measuring social inequalities in health [17] and has been referred to as a "workhorse in most health economic studies" [18]. The CI measures socioeconomic inequalities in health by taking into account individuals' level of health and rank in the socioeconomic domain [19]. The Gini coefficient (GC) measures socioeconomic inequalities in income by taking into account individuals' income level and income rank. As indicated in the Data and Methods section, the CI and the GC are virtually identical in that they have the same formula (except for some differences in the attributes that they attempt to address).

The present paper focuses on income-related inequalities in health in Korea. Specifically, the paper estimates socioeconomic inequalities in income and income-related inequalities in health for the whole population as well as within/across regions by using the GC and the CI, respectively. In addition, the paper examines the relationship between these inequalities across regions. Based on the results, the paper examines whether socioeconomic inequalities in health across regions would converge (given a convergence of regional income to a higher level of income) and provides policy implications for the more equitable distribution of health.

## Data and methods

The data were drawn from the Korea National Health and Nutrition Examination Survey (KNHANES), a comprehensive and representative survey conducted every three years by the Korea Center for Disease Control and Prevention (KCDC) to assess the health of the Korean population. The survey uses household registries to collect data from a stratified multistage probability random sample based on geographic regions, administrative districts, and types of residences. KNHANES provides demographic, socioeconomic, and dietary information as well as medical history information collected via personal interviews. Four waves (1998, 2001, 2005 and 2007) of KNHANES surveys are available. The first three surveyed samples over a couple of months during the survey year. However, the fourth wave employed three independent samples and extended the survey period to the entire year for all three years by considering one circulatory sample for each year. Hence, the data from the 2007 survey covered only one fifth of the sample surveyed in the fourth wave. KCDC has yet to announce future data releases [20]. We considered a total of 45,233 subjects who were at least 19 (Table 1).

**Table 1:** Distribution of gender and waves by region

Region		male	female	1998	2001	2005	2007	Total	Share^1)^	Si/Do^2)^
Gangwon	N	717	828	304	296	842	103	1545		Do
	%	1.59	1.83	0.67	0.65	1.86	0.23	3.42	3.1	
Gyeonggi	N	3868	4371	1453	1510	4726	550	8239		Do
	%	8.55	9.66	3.21	3.34	10.45	1.22	18.21	22.03	
Gyeongnam	N	1437	1729	838	465	1632	231	3166		Do
	%	3.18	3.82	1.85	1.03	3.61	0.51	7	6.46	
Gyeongbuk	N	1201	1474	626	379	1415	255	2675		Do
	%	2.66	3.26	1.38	0.84	3.13	0.56	5.91	5.52	
Gwangju	N	634	733	230	296	730	111	1367		Si
	%	1.4	1.62	0.51	0.65	1.61	0.25	3.02	3	
Daegu	N	1026	1281	464	411	1249	183	2307		Si
	%	2.27	2.83	1.03	0.91	2.76	0.4	5.1	5.21	
Daejeon	N	606	709	176	254	789	96	1315		Si
	%	1.34	1.57	0.39	0.56	1.74	0.21	2.91	3.05	
Busan	N	1658	1996	740	626	2106	182	3654		Si
	%	3.67	4.41	1.64	1.38	4.66	0.4	8.08	7.45	
Seoul	N	4242	5011	1749	1768	5239	497	9253		Si
	%	9.38	11.08	3.87	3.91	11.58	1.1	20.46	20.77	
Ulsan	N	436	496	90^3)^	194	591	57	932		Si
	%	0.96	1.1	0.2	0.43	1.31	0.13	2.06	2.22	
Incheon	N	1011	1145	339	398	1276	143	2156		Si
	%	2.24	2.53	0.75	0.88	2.82	0.32	4.77	5.35	
Jeonnam	N	912	1091	503	309	1026	165	2003		Do
	%	2.02	2.41	1.11	0.68	2.27	0.36	4.43	3.85	
Jeonbuk	N	1026	1226	469	403	1291	89	2252		Do
	%	2.27	2.71	1.04	0.89	2.85	0.2	4.98	3.77	
Jeju	N	413	460	94	154	551	74	873		Do
	%	0.91	1.02	0.21	0.34	1.22	0.16	1.93	1.13	
Chungnam	N	886	1030	527	304	948	137	1916		Do
	%	1.96	2.28	1.17	0.67	2.1	0.3	4.24	4	
Chungbuk	N	736	844	389	299	785	107	1580		Do
	%	1.63	1.87	0.86	0.66	1.74	0.24	3.49	3.09	

Total	N	20809	24424	8991	8066	25196	2980	45233		
	%	46	54	19.88	17.83	55.7	6.59	100		

1) Each region's share of Korea's population based on the national census in 2005.

2) Si and Do denote autonomous metropolitan cities and provinces, respectively.

3) Subjects for Ulsan for 1998 were re-coded from the initial classification of Gyeongnam.

We adjusted household income by using a consumer price index (CPI = 100 in 2005) for each region and calculated per capita income by dividing CPI-adjusted household income by family size. Those subjects providing no information on their income (n = 1,460) were omitted, and the remaining 43,773 observations were used for the estimation of the GC and the CI and for the regression analysis for establishing the correlation between inequalities and income.

We assessed the subjects' health based on self-rated health (SRH). We asked the subjects a question about their health ("How would you rate your current health compared to that of others of your age?"), and they rated their health on a scale ranging from ① very healthy" to ⑤ very unhealthy." We considered true health is a continuous latent variable following a standard lognormal distribution underlying the self-rated health status. We obtained ill-health scores for each category by matching the cumulative sample proportion to the probability of the standard lognormal distribution. To control for the effects of the subjects' age and gender, we standardized the raw scores indirectly by substituting age/gender average scores (excluding the subject's own score) for individual scores [6].

We assessed the extent of inequalities in income and health by using the GC and the ill-health CI, respectively. The GC, a popular measure of inequalities in income, refers to the ratio of the area that lies between the line of equality and the Lorenz curve, which plots the cumulative proportion of the total income of the population to the cumulative proportion of population (beginning with the lowest income group or individual). We used the CI to summarize income-related inequalities in health and measured these inequalities by the area between the line of equality and the ill-health concentration curve, which displayed the cumulative proportion of ill-health to the cumulative proportion of the population by per capita income (beginning with the most disadvantaged populations). This CI ranged from positive to negative values depending on whether the curve fell below or above the diagonal, respectively. If the CI was 0, then we assumed perfect equality. If CI > 0 (or < 0), then we assumed that ill-health were concentrated in the highest (or lowest) socioeconomic groups. The absolute value of the CI indicated the extent of income-related inequalities in health.

The GC and the CI were obtained as follows:

$$C=\frac{2}{n\bar{x}}\overset{n}{\underset{i=1}{\sum }}x_{i}R_{i}-1,$$

where $\bar{x}$ denotes the average income and the average ill-health scores for the GC and the CI, respectively, and *R_i_*indicates the relative ranking of the i-th individual/group/region (beginning with the individual/group/region with the lowest income). We calculated the inequalities in income and health across regions by using C and called these inequalities "spatial inequalities."

Although standard errors associated with the GC and the CI can obtained using various assumptions and methods [7,21-24], they are seldom reported because of mathematical difficulties or heavy computational burdens [22]. In this study, we employed bootstrapping, a distribution-free simulation-based method, to estimate the CI and its confidence intervals. We obtained the GC/CI percentiles from bootstrapping with 1,000 replications and employed the t-test to examine gender differences in health scores. Further, we conducted a regression analysis with appropriate weights to evaluate the relationships among the level of income, income inequalities, and socioeconomic inequalities in health. We used SAS Version 9.0 (SAS Institute Inc., Cary, NC, USA) for all the analyses.

## Results

Tables 1, 2, 3 and 4 present the subjects' gender, mean age, and income as well as the share of older individuals and the waves by region. The results indicate that 46.4% of the subjects resided in cities (Si), which is consistent with the national average (47.5%) (Table 1). Jeonnam was the oldest province, with the mean age of 51.8 ± 16.7 (SD), whereas Ulsan was the youngest, with the mean age of 41.5 ± 14.4 (Table 2). Specifically, 26.6% and 16.4% of the subjects in Jeonnam were 65 and above and 70 and above, respectively, whereas only 8.7% of those in Ulsan were 65 and above (Table 2). Ulsan showed the highest average income, whereas Jeonnam, the lowest (Table 3).

**Table 2:** Mean age and proportion of older individuals by region

Region				Elderly, ≥ 65		Elderly, ≥ 70	
				
	N	average	SD	N	%	N	%
Gangwon	1545	49.1	15.9	299	19.4	160	10.4
Gyeonggi	8239	43.2	15.0	949	11.5	567	6.9
Gyeongnam	3166	45.5	15.9	465	14.7	291	9.2
Gyeongbuk	2675	50.2	17.1	651	24.3	423	15.8
Gwangju	1367	43.3	15.8	159	11.6	103	7.5
Daegu	2307	43.7	15.6	270	11.7	158	6.8
Daejeon	1315	44.6	15.4	170	12.9	87	6.6
Busan	3654	44.0	15.3	408	11.2	233	6.4
Seoul	9253	42.9	15.3	955	10.3	543	5.9
Ulsan	932	41.5	14.4	81	8.7	46	4.9
Incheon	2156	43.0	15.0	242	11.2	149	6.9
Jeonnam	2003	51.8	16.7	532	26.6	328	16.4
Jeonbuk	2252	47.6	16.6	413	18.3	248	11.0
Jeju	873	46.4	16.5	140	16.0	88	10.1
Chungnam	1916	48.9	16.9	441	23.0	247	12.9
Chungbuk	1580	47.0	16.4	274	17.3	163	10.3

Total	45233	45.0	15.9	6449	14.3	3834	8.5

SD: Standard deviation.

**Table 3:** Average per capita income by region

Region	N	Income	SD	rank
Gangwon	1519	61.7	50.4	11
Gyeonggi	7924	71.9	51.0	3
Gyeongnam	3051	59.6	48.8	14
Gyeongbuk	2544	59.3	54.6	15
Gwangju	1336	63.8	48.3	8
Daegu	2213	61.5	45.1	12
Daejeon	1265	70.0	44.9	4
Busan	3599	61.2	44.4	13
Seoul	8899	79.3	58.9	2
Ulsan	911	83.6	58.4	1
Incheon	2109	68.9	48.7	5
Jeonnam	1947	53.8	50.2	16
Jeonbuk	2239	64.3	48.3	7
Jeju	852	64.5	48.4	6
Chungnam	1832	62.5	57.1	10
Chungbuk	1533	63.2	54.7	9

Total	43773	67.8	52.6	

SD: Standard deviation.

Income measured in 10,000 KRW.

**Table 4:** Self-rated health by region

Region	very healthy	healthy	fair	unhealthy	very unhealthy	Total
Gangwon	85(5.50)	511(33.07)	552(35.73)	339(21.94)	58(3.75)	1545
Gyeonggi	373(4.53)	3528(42.82)	2902(35.22)	1238(15.03)	198(2.40)	8239
Gyeongnam	127(4.01)	1184(37.4)	1103(34.84)	622(19.65)	130(4.11)	3166
Gyeongbuk	83(3.10)	811(30.32)	939(35.10)	727(27.18)	115(4.30)	2675
Gwangju	107(7.83)	498(36.43)	519(37.97)	192(14.05)	51(3.73)	1367
Daegu	108(4.68)	764(33.12)	876(37.97)	456(19.77)	103(4.46)	2307
Daejeon	61(4.64)	504(38.33)	510(38.78)	213(16.20)	27(2.05)	1315
Busan	162(4.43)	1295(35.44)	1419(38.83)	661(18.09)	117(3.20)	3654
Seoul	513(5.54)	3777(40.82)	3309(35.76)	1403(15.16)	251(2.71)	9253
Ulsan	42(4.51)	338(36.27)	375(40.24)	147(15.77)	30(3.22)	932
Incheon	130(6.03)	894(41.47)	764(35.44)	315(14.61)	53(2.46)	2156
Jeonnam	88(4.39)	735(36.69)	624(31.15)	472(23.56)	84(4.19)	2003
Jeonbuk	118(5.24)	845(37.52)	797(35.39)	420(18.65)	72(3.20)	2252
Jeju	52(5.96)	333(38.14)	293(33.56)	168(19.24)	27(3.09)	873
Chungnam	60(3.13)	645(33.66)	678(35.39)	444(23.17)	89(4.65)	1916
Chungbuk	56(3.54)	655(41.46)	537(33.99)	273(17.28)	59(3.73)	1580

Total	2165(4.79)	17317(38.28)	16197(35.81)	8090(17.89)	1464(3.24)	45233

Numbers in parentheses indicate percentages.

Table 4 shows the distribution of SRH by region. Gwangju had the highest percentage of very healthy subjects (7.83%), and Gyeongbuk, the lowest (3.10%). On the other hand, Chungnam had the highest percentage of very unhealthy subjects, whereas Daejeon, the lowest (2.05%). Overall, 4.79% of all subjects responded that they were very healthy, and 3.24%, very unhealthy.

**Figure 1:**
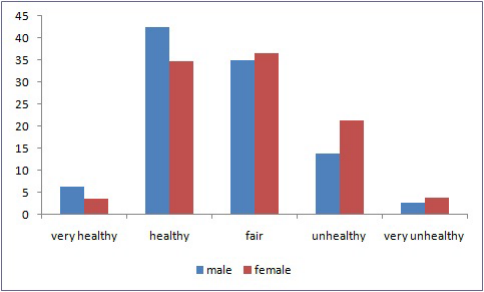
**Self-reported health distribution by gender**.

**Figure 2:**
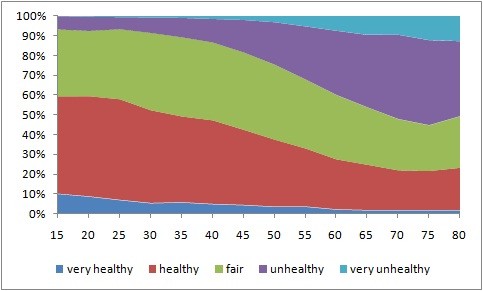
**Self-reported health distribution by age group**. The x-axis: Five-year age groups.

**Figure 3:**
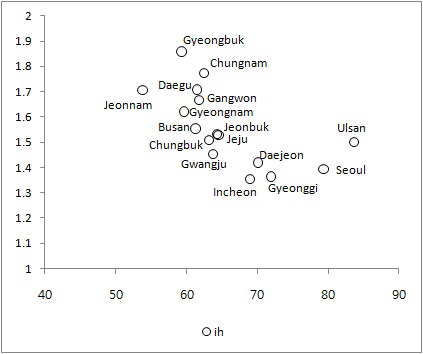
**Average ill-health scores plotted against average regional income for 16 regions**. The x-axis: Per capita income. The y-axis: Ill-health scores.

Figure 1 shows gender differences in health, and Figure 2 shows SRH scores by age group. The results indicate substantial differences in health. Male subjects were healthier than their female counterparts. Further, younger subjects were healthier than older ones. Figure 3 shows the average ill-health scores plotted against average regional income for 16 regions. High-income regions were healthier than low-income ones.

**Figure 4:**
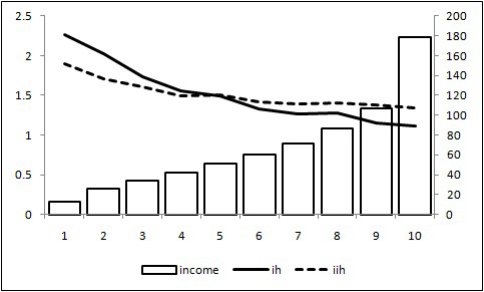
**Average (raw and standardized) ill-health scores by income group**. ih (left): Average ill-health scores for each income group. iih (left): Average ill-health scores standardized indirectly. income (right): Real per capita income.

Figure 4 shows the average (raw and standardized) ill-health scores by income group. The regression line for the standardized ill-health score was (*p *= 0.0125):

$$ill-health=1.6937-0.0265\times income,R^{2}=0.5620.$$

The results indicate that high-income groups were healthier than low-income ones, which is consistent with the findings of previous studies demonstrating the positive income elasticity of health [2,6-18].

Table 5 summarizes the inequality measures for various cases. The upper part of Table 5 shows the overall inequalities in income (GC) and health (CI, CI*) for the whole sample. The lower part indicates the inequalities in income (GC) and health (CI, CI*) across 16 regions. Here CI* denotes the CI obtained from gender/age-adjusted ill-health scores based on the indirect standardization in Kakwani et al [6]. The GC was 0.3763 for overall and 0.0657 for spatial inequalities. The income-related CI was -0.1309 for overall and -0.0473 for spatial inequalities. The overall GC ranged from 0.356 to 0.407 across waves, and the CI, from -0.1011 to -0.1484.

**Table 5:** Estimates of overall and spatial inequalities in income and health

	Percentile	GC	CI	CI*
Overall inequalities	estimate	0.3763	-0.1309	-0.0589
	0.005	0.3723	-0.1378	-0.0617
	0.025	0.3733	-0.1365	-0.0609
	0.05	0.3738	-0.1356	-0.0606
	0.5	0.3763	-0.1308	-0.0589
	0.95	0.3788	-0.1265	-0.0571
	0.975	0.3791	-0.1255	-0.0568
	0.995	0.3800	-0.1239	-0.0561

Spatial inequalities	estimate	0.0657	-0.0473	-0.0282
	0.005	0.0624	-0.0484	-0.0291
	0.025	0.0629	-0.0482	-0.0290
	0.05	0.0631	-0.0481	-0.0289
	0.5	0.0646	-0.0466	-0.0279
	0.95	0.0654	-0.0451	-0.0272
	0.975	0.0655	-0.0448	-0.0271
	0.995	0.0656	-0.0441	-0.0268

Spatial GC/CI rankings are based on regional average income.

Spatial inequalities indicate the GC and the CI calculated across regions.

Percentiles were obtained through bootstrapping simulations with 1,000 replications.

CI: The CI of ill-health scores.

CI*: The CI of indirectly standardized ill-health scores by age and gender.

GC: The Gini Coefficient.

Table 6 shows the within-region inequalities in income and health for 16 regions. Gyeongbuk showed the highest ill-health (1.859), and Incheon, the lowest ill-health (1.355). The relationship between ill-health scores and income was estimated across 16 regions by using the data as follows (*p *< 0.001):

**Table 6:** Inequalities in income and health within regions

Region	Income	Ill-health	GC	CI	CI*	N
Gangwon	61.7	1.667	0.4008	-0.1479	-0.0732	1519
Gyeonggi	71.9	1.364	0.3488	-0.1215	-0.0497	7924
Gyeongnam	59.6	1.621	0.3914	-0.1215	-0.0631	3051
Gyeongbuk	59.3	1.859	0.4124	-0.1192	-0.0766	2544
Gwangju	63.8	1.454	0.3745	-0.1187	-0.0282	1336
Daegu	61.5	1.709	0.3555	-0.1410	-0.0438	2213
Daejeon	70.0	1.420	0.3199	-0.1151	-0.0254	1265
Busan	61.3	1.554	0.3535	-0.0981	-0.0439	3599
Seoul	79.3	1.393	0.3655	-0.1234	-0.0423	8899
Ulsan	83.6	1.501	0.3504	-0.0844	-0.0407	911
Incheon	68.9	1.355	0.3550	-0.1293	-0.0575	2019
Jeonnam	53.8	1.707	0.4231	-0.1576	-0.0846	1947
Jeonbuk	64.3	1.533	0.3753	-0.1136	-0.0538	2239
Jeju	64.5	1.528	0.3504	-0.1596	-0.0498	852
Chungnam	62.5	1.773	0.4233	-0.1209	-0.0634	1832
Chungbuk	63.2	1.508	0.4086	-0.1076	-0.0635	1533

Income: The region's average per capita income.

Ill-health: Average ill-health scores.

CI: The CI of ill-health scores.

CI*: The CI of indirectly standardized ill-health scores by age and gender.

GC: The Gini Coefficient.

$$ill-health=2.5215-0.1482\times income,R^{2}=0.5855.$$

The regression estimates indicate a significant relationship between ill-health scores and income across regions. High-income regions were healthier than low-income ones. Further, income level accounted for more than half of the total variation in ill-health scores across income groups and regions. Both regressions had considerable explanatory power even when we did not control for other health factors such as socioeconomic status, regional characteristics, health resources, and health behaviors, which are well summarized in Fang et al [25].

**Figure 5:**
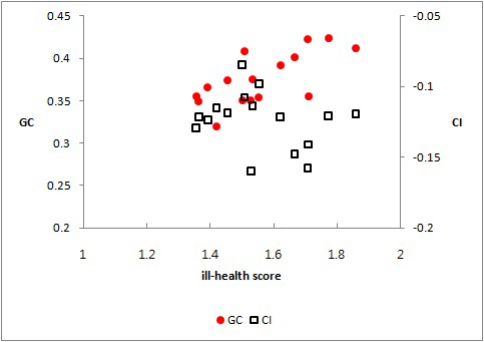
**The GC (left) and the CI (right) plotted against ill-health scores**. The y-axis (left): The Gini coefficient. The y-axis (right): The Concentration Index. The x-axis: Ill-health scores.

Figure 5 plots the within-region GC and the within-region CI against ill-health scores for 16 regions. The GC was positively related to ill-health, whereas the CI was negatively related. An increase in the average ill-health increased income and health inequalities across regions. Ill-health scores were positively correlated with income and health inequalities.

**Figure 6:**
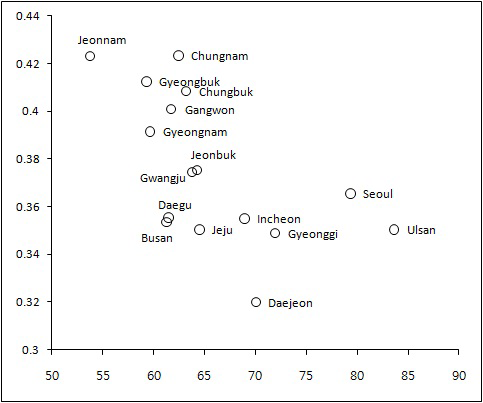
**Within-province Gini coefficient and average income for 16 regions**. The x-axis: Per capita income. The y-axis: The Gini Coefficient.

Table 7 shows the relationship between health inequalities and the level of level/income inequalities for 16 regions. As shown in Figure 6 (the scatter plot), the GC was negatively related to the level of income. The CI was positively related to the level of income, but the GC was negatively related. In particular, the results indicate a strong relationship between gender/age-adjusted inequalities in health (CI*) and the average income as well as income inequalities (GC) with large R^2^. An increase in the region's average income reduced income inequalities and income-related inequalities in health.

**Table 7:** Regression estimates of health inequalities across regions

**dep. var**.	intercept	GC	income	R^2^
GC	0.4945		-0.0181	0.304
	(< .0001)		(0.0268)	
CI	-0.1510		0.0042	0.051
	(0.0004)		(0.3985)	
CI*	-0.1248		0.0107	0.385
	(0.0002)		(0.0104)	
CI	-0.0661	-0.1518		0.072
	(0.2425)	(0.3143)		
CI*	0.1051	-0.4237		0.655
	(0.0041)	(0.0001)		

Values in parentheses indicate the p-values of the estimates directly above.

CI: The CI of ill-health scores.

CI*: The CI of indirectly standardized ill-health scores by age and gender.

GC: The Gini Coefficient.

## Discussion

In addition to inequalities at the country level, those at the regional level reflect a major source of concern because they can trigger regional conflicts and thus destabilize the country. Together with the rapidly aging population and economic crises, inequalities in income and health both within and across regions have emerged as an important issue in Korea. Thus, finding ways to effectively reduce such inequalities has become an urgent task.

In general, individuals' gender and age are important indicators of their health. Male subjects were healthier and had fewer health problems than their female counterparts. These results demonstrate gender differences in the health of individuals (Figure 1). The average ill-health score was 1.3417 for male subjects and 1.6657 for female subjects (*p *< .0001). For all the waves, male subjects were healthier than their female counterparts.

There were substantial differences in the age of subjects across regions. The mean age varied from 41.5 for Ulsan to 51.8 for Jeonnam (Table 2). Older individuals (those 65 and over) accounted for more than 20% of the subjects in Gyeongbuk, Jeonnam, and Chungnam, whereas they accounted for only 8.7% and 10.3% of the subjects in Ulsan and Seoul, respectively. In particular, Gyeongbuk and Jeonnam accounted for more than 15% of those subjects 70 and above (Table 2).

The results indicate a close relationship between the subjects' age and health inequalities (Figure 2). Older individuals are more likely to have health problems and require health-care services than younger ones. Thus, without proper attention and care, age differences can lead to substantial health inequalities across the country as well as within/across regions. In this regard, Korea's rapidly aging population can accelerate health inequalities in the country, and thus, there is an urgent need for proper attention.

When dealing with health inequalities, socioeconomic factors represent a source of major policy concern. Jeonnam had the lowest per capita income, and Ulsan, the highest. Jeonnam was the oldest and the poorest, whereas Ulsan, the youngest and the richest. There were clear differences in the subject's age and income across regions (Tables 2 and 3).

The ill-health scores were lower for high-income groups than for low-income ones (Figure 4). The regression results indicate a significant negative relationship between ill-health scores and income. The level of income accounted for 56.2% of the total variation in ill-health.

The comparison of the subjects' health status across regions provides similar results. Ill-health scores were negatively related to the average income across regions (Figure 3). High-income regions were healthier than low-income ones (*p *< 0.001, *R*^2 ^= 0.5855). Both the overall and spatial regression estimates indicate a significant negative relationship between ill-health and income. These regressions explained more than half of the total variation in ill-health across income groups and regions.

These findings were independent of gender/age adjustment. For example, high-income groups and regions were healthier than low-income ones regardless of whether we controlled for gender and age effects, leaving a large portion of health status to be explained by socioeconomic and behavioral factors.

There was a strong relationship between gender/age adjusted health and income inequalities (Table 7). In particular, the absolute value of the CI* increased when the GC increased. Income and health inequalities moved in the same direction, suggesting that a decrease in income inequalities may reduce socioeconomic health inequalities.

The overall GC was 0.376 (Table 5), which was slightly larger than those (0.31-0.32) reported by Kim et al [26]. The spatial GC was 0.0657, which was smaller than the overall GC but consistent with Park and Yu's [27] estimates ranging from 0.06-0.12. The smaller spatial inequalities may be due to the existence of sizable within-region inequalities.

All the CI values were negative, indicating that disadvantaged individuals and regions were less likely to be healthy. This result is consistent with the findings of previous studies [6,7]. The overall health inequality estimate was -0.1308, and the age/gender-adjusted health inequality estimate was -0.059 (Table 5). The size of income-related health inequalities in Korea was similar to those of the Netherlands, Spain, and Switzerland but smaller than those for the U.S. and the U.K. [7]. The spatial CI was -0.0473, and the age/gender-adjusted CI was -0.0282. The spatial inequalities in health (like income inequalities) were smaller than the overall inequalities. This may be because we averaged the within-region variations in obtaining the special inequalities. As shown in Table 6 there were substantial income-related inequalities in health within regions (the CI ranged from -0.084 to -0.1596). In this regard, when devising and implementing policy initiatives to reduce regional inequalities in income and health, it is recommended for policymaker to take into account within-region variations.

These results are consistent with the findings of previous studies considering different data sources. Previous study considering the Korea Labor Panel has reported various socioeconomic (education, job, and income) inequalities in mortality. Kang [2] have found regional inequalities in mortality, demonstrating that high-income regions show lower mortality than low-income regions.

Both income and health inequalities were related to the level of health and income. The regions with poor average health were more likely to show large income inequalities and health inequalities than those with better health (Figure 5). Further, the GC was larger for low-income regions than for high-income ones (Figure 6). These results are consistent with the findings of previous studies demonstrating that groups/states with low quality of health are more likely to show large health inequalities. For example, Native Americans/Native Alaskans had the lowest quality of health and faced the largest overall and income-related inequalities in health, and Kentucky and West Virginia had lowest quality of health and showed the largest inequalities in health [9].

In addition, there were strong relationships among the CI, the GC, the level of income, and health status. The regression estimates indicate a negative relationship between the GC and the level of income; a positive relationship between the CI and the level of income; and a negative relationship between the CI and the GC (Table 7). The higher the region's average income, the smaller the region's income inequalities were. Further, an increase in the region's average income reduced its inequalities in health. This suggests that an increase in a region's average income reduces its income inequalities and that this leads to a more equitable distribution of health. Further, the larger a region's income inequalities, the larger the region's health inequalities are and vice versa.

Finally, the results reveal a possibility of convergence of socioeconomic inequalities in health across regions. Previous studies of income convergence across regions in Korea have produced mixed results. Some studies have found regional income convergence [28,29], noting a decreasing trend since the Asian financial crisis of 1997 [30], whereas others have provided no conclusive findings [31] or suggested the possible divergence of regional economies since the crisis [27]. In general, previous studies have indicated that regional income converged before the crisis but that it deteriorated or stagnated after the economic crisis. In the present study, both health and income inequalities were related to average per capita income, which suggests that these inequalities may show similar patterns. In sum, given a convergence of regional income to a higher level, regional health levels may follow a similar path, and health inequalities within/across regions may decrease.

## Conclusions

Health inequalities have become an urgent social issue in many parts of the world, including advanced countries. For example, in the U.S., the Obama administration implemented an intense policy initiative to push government funded health insurance by stressing various negative effects of unequal access to health-care services on American health. Thus, the urgent need for reducing health inequalities across income groups and/or regions applies to all countries, including Korea.

The results indicate substantial socioeconomic inequalities in health both within and across regions. Previous studies have justifiably stressed the need for reducing huge inequalities across regions, but the results of the present study suggest the importance of reducing inequalities in health not only across regions but also within regions. Thus, although regional inequalities require proper attentions, policy initiatives should also focus on within-region variations in the distribution of health.

In terms of policy initiatives focusing on the welfare of the public, it is crucial to achieve economic growth while reducing socioeconomic inequalities in income and health within/across regions. However, since proportional growth, which leaves income inequalities unchanged, could lead to greater health inequalities [16], reducing socioeconomic health inequalities may require more progressive policy initiatives.

Any efforts to strike a balance between health equality and income growth should be based on long-term social goals, which is precisely the reason why there is an urgent need for assessing the current distribution of health. In this regard, future research should consider a wide range of health factors, regional characteristics, and policy objectives to better predict future inequalities in health both within and across regions.

In addition, this study is based on pooled cross-sectional data, which make it difficult to determine causal relationships between health indicators and health-related factors. Therefore, longitudinal studies should be helpful for inferring the convergence of health inequalities across regions.

## Competing interests

The authors declare that they have no competing interests.

## Authors' contributions

All the authors contributed to this research. BCA (the main author) was responsible for analyzing the data and prepared the manuscript. EJH participated in interpreting the results and revising the manuscript.

This manuscript was approved by all the authors.
